# Corpus luteum and progesterones in embryo transfer cycles: current challenges of different luteal phase support protocols

**DOI:** 10.5935/1518-0557.20240044

**Published:** 2024

**Authors:** Maria do Carmo Borges de Souza, Roberto de Azevedo Antunes, Marcelo Marinho de Souza, Hitomi Miura Nakagawa, Adelino Amaral Silva, Emerson Barchi Cordts, Caio Parente Barbosa

**Affiliations:** 1 Fertipraxis Centro de Reproducao Humana, Rio de Janeiro, RJ, Brazil; 2 Genesis - Centro de Assistencia em Reproducao Humana, Brasília, DF, Brazil; 3 Instituto Idéia Fértil, and Embryo Genesis Reproducao Humana - São Paulo, SP, Brazil

The proposed indications for the use of progesterone encompass a broad spectrum of medical scenarios, including hormone replacement therapy in menopause, addressing infertility due to luteal phase deficiency, managing the threat of miscarriage and recurrent miscarriages, treating menstrual disorders, alleviating symptoms of endometriosis, and supporting Assisted Reproductive Techniques (ART). Notably, progesterone is now integrated into Progesterone-primed ovarian stimulation cycles, as elucidated by (Kuang *et al*., 2015). The recognition of luteal phase insufficiency as a potential issue traces back to Noyes *et al*. (1950). As Kliman (2020) highlighted, understanding the intricate developmental changes within the endometrium throughout the menstrual cycle remains pivotal. Currently, the need for progesterone supplementation in ART cycles, encompassing fresh and various types of frozen embryo transfer (FET) protocols, is firmly established (Carp, 2020). The latter transfer method’s prevalence is noteworthy, representing 66.6% of all transfers in Latin America (Zegers-Hochschild *et al*., 2023) and 56.8% globally (Adamson *et al*., 2018).

Regarding live birth rates (LBR), clinical frontiers remain in the luteal phase support (LPS) in ART cycles. Over the past decade, significant research efforts have delved into various LPS protocols, aiming to assess their efficacy in terms of implantation rates, clinical pregnancies, live birth rates, and rates of spontaneous abortion (van Der Linden *et al*., 2015). Additionally, thorough evaluations of safety profiles, side effects, patient compliance, and costs have been undertaken, as highlighted by Pabuccu *et al*. (2022). Despite the absence of expectations for premature luteolysis in Frozen Embryo Transfer (FET) cycles due to controlled ovarian stimulation, choices regarding early LPS remain subject to debate.

Different LPS formulations, when to start, the best route of administration, dosage and duration, and whether there is a place for additional agents are frequently discussed in the literature. The latest ESHRE guidelines on ovarian stimulation, after assessing the best available evidence at that time, pointed out that any of the non-oral natural progesterone could be used as LPS. At the same time, dydrogesterone (DYD) was recommended under strong conditional evidence from large RCTs as the only oral LPS alternative to have the same ongoing pregnancy rates (OPR) probably and to show similar safety and tolerability as other types of natural progesterones (The ESHRE Guideline Group on Ovarian Stimulation, 2020). An important aspect to emphasize is that while DYD is categorized separately from other natural progesterones, it is derived from a natural source (discorea villosa - Mexican yam). Developed in the 1960s, this compound transforms when exposed to ultraviolet light into a retroprogesterone biochemical configuration, enabling its efficacy via the oral route (Barbosa *et al*., 2018).

In fresh embryo transfer cycles, the most prescribed LPS protocols, utilizing either micronized progesterone (MP) administered vaginally or DYD orally, have been extensively compared in double-blind, placebo-controlled, randomized controlled trials (RCTs). Notably, studies conducted by Tournaye *et al*. (2017) and Griesinger *et al*. (2018) have demonstrated comparable outcomes in terms of OPR, LBR, and safety profiles between these two protocols. However, recent findings from an individual participant data meta-analysis (Griesinger *et al*., 2020) revealed a significant difference favoring oral DYD over vaginal MP, with a 16% increase in OPR and a 19% increase in LBR associated with DYD usage. This, plus the high oral bioavailability (28% more bioavailable versus 5% compared to MP) of DYD and a much friendlier tolerability and side effects profile, presents DYD as a potential first choice for LPS in fresh embryo transfer cycles.

As previously discussed, the predominant trend in contemporary embryo transfer cycles revolves around FET cycles. This shift is globally observed, driven by the adoption of diverse freeze-all policies, widespread implementation of measures to mitigate ovarian hyperstimulation, and a rising number of preimplantation genetic tests conducted (Zegers-Hochschild *et al*., 2023). Various FET protocols are outlined, typically categorized as artificial cycles (AC) or natural cycles (NC), differing primarily in the presence or absence of a corpus luteum to aid LPS. Although overall CPR appears comparable between AC and NC protocols (Glujovsky *et al*., 2020), emerging evidence suggests a potential association between AC FET and elevated risks of preterm births and preeclampsia (Zaat *et al*., 2023). So far, considerable debate and controversy persist regarding the optimal LPS approach for different FET strategies.

AC FET is still the primary strategy used by most clinicians. This occurs because artificial cycles are more convenient and flexible when scheduling ET. There is a debatable lower cancelation rate when LPS in AC is started after estrogen priming. Different estrogen supplementation dosages and routes have similar results in achieving an endometrium of at least 7mm (Madero *et al*., 2016). When it comes to progesterone supplementation, natural progesterones present similar OPR, miscarriage rates (MR), and LBR outcomes through different routes of administration, whether vaginally or intramuscular (Abdelhakim *et al*., 2020). Also, there is some evidence regarding similar results using natural progesterone rectally (Alsbjerg *et al*., 2023) or subcutaneously (Conforti *et al*., 2021). The only oral progesterone with similar OPR, MR, and LBR and a good safety profile used for AC FET cycles is DYD (Pabuccu *et al*., 2022).

A recently proposed personalization of AC FET considers progesterone serum levels on the day of the embryo transfer after micronized vaginal progesterone (MVP) use. Progesterone levels lower than 8.8 - 9.2ng/mL were aligned with significantly lower OPR (36.6% x 54.4%) and CPR (35.5% x 52.0%) (Labarta *et al*., 2021). There are already several proposed rescue strategies for LPS when low serum progesterone levels are detected, including the use of DYD (Mackens *et al*., 2023), intramuscular or subcutaneous MP (Álvarez *et al*., 2021).

Since AC FET has been associated with poorer obstetrical outcomes, NC FET has increased (Zaat *et al*., 2023). NC FET encompasses various protocols, including truly NC without LPS, with LPS, modified NC with an hCG trigger, or even with ovarian stimulation agents, such as letrozole, for example. All types of NC FET present similar OPR, MR, and LBR (Glujovsky *et al*., 2020), except for truly NC without LPS (Mizrachi *et al*., 2021). This highlights the importance of LPS, even in NC FET. So far, there are no differences regarding clinical outcomes or whether LPS is performed with DYD or MP (Jiang *et al*., 2023).

Vaginal administration was the preferred route for luteal phase support in a physician’s questionnaire survey (Di Guardo *et al*., 2020) that also detected an emerging use of the oral route as well as the continuation of luteal support until 12 weeks of gestation. More than half of the clinicians surveyed adhered to this practice, highlighting the difference between evidence-based medicine and real-life practice.

Recent publications have addressed congenital malformations in babies born through Assisted Reproductive Technology (ART), with a potential link to DYD. One of the first papers to mention the positive association between DYD usage in early pregnancy and the risk of developing heart disease in offspring was conducted by Zaqout *et al*. (2015). This group retrospectively studied 202 children born with congenital heart disease, compared to a study control group of 200 children born free of heart abnormalities from 2010 to 2013. Binary logistic regression analyses used to analyze a potential relationship between drug exposure and congenital heart disease found that mothers of children born with congenital heart disease received more DYD during the first trimester of pregnancy than mothers from the control group [adjusted odds ratio 2.71 (95% CI 1.54-4.24); *p*=0.001].

Although the cited paper was a retrospective control study, the mothers conceived spontaneously without being appropriately selected for clinical history; the number of cases was limited, and a possible bias related to the reason why the patients used DYD was not enlightened; the published results raised justified concerns, once congenital heart disease is the most frequent abnormality in newborns; genetics explains less than 20% of the cases and the resulting burden on the health of the affected children can be rather severe.

Some authors investigated an association with DYD exposure during ART cycles in an attempt to find non-inherited risk factors that might explain etiologies for congenital heart disease other than genetic ones. Notably, Zaqout *et al*. (2015) received criticism for failing to adhere to scientific principles in epidemiological research, including issues with the case base, de-confounding, and comparable accuracy. The latest publication by Henry *et al*. (2023) fails to establish any definitive causal relationships, as the author recognized such limitation in the abstract of his paper.

Also, a review by Koren *et al*. (2020) analyzed the fetal safety of all medications used in treating infertility, including clomiphene citrate, aromatase inhibitors, metformin, gonadotropins, and progestins. The authors addressed congenital malformations in babies born through Assisted Reproductive Technology (ART) since the rates of congenital malformations among infertile women giving birth and conceiving spontaneously were higher than the rates among healthy women conceiving spontaneously. They reported a potential link to DYD. This paper was retracted for not adhering to scientific principles in epidemiological research.

It is estimated that between 1977 and 2005, around 10 million pregnancies were treated with DYD. Yet, until 2019, only a few studies, with a total sample size of less than 600, contained reports on fetal safety. It is not easy to establish an association with drug exposure, considering female infertility itself can be a significant reason for a higher incidence of newborn malformations; indeed, the majority of clinical ART studies do not report pregnancy outcomes or neonatal events, once the primary endpoint of most of them is success in inducing pregnancy.

A systematic review and meta-analysis by Saccone *et al*. (2017), which included only randomized clinical trials, reported data from progestogen supplementation in the first trimester of pregnancy to prevent miscarriage in women with unexplained recurrent abortions. No statistically significant differences were found for fetal mortality (RR 1.80, 95% CI 0.44-7.34) or abnormalities (RR 1.68, 95% CI 0.22-12.62), raising evidence for the benefits of progestins to prevent miscarriage, being safe for the fetuses.

Progesterones have been used for more than 60 years. There is no data to support the suggestion that first-trimester use increases the risk of fetal abnormalities, including DYD (Griesinger *et al*., 2020; Katalinic *et al*., 2022). Additional information must be added to provide more definitive answers on the best LPS protocols for embryo transfer. As Berntsen *et al*. (2019) pointed out, the debate must include ART and concerns about the procedures per se, not just medications. Attention to the surveillance of ART children by ART registries and national health care registries remains paramount. Extensive clinical studies of ART children are necessary to provide more consistent data on the consequences to health and the underlying mechanisms responsible for the possible negative influence of ART in general.
